# The FRED‐X Flow Diverter—An Australian Experience

**DOI:** 10.1111/1754-9485.13857

**Published:** 2025-04-14

**Authors:** Peter Shuangyue Tan, Charles Li, Cameron Williams, Winston Chong, Carlos Chung, Conor Houlihan, Anoop Madan

**Affiliations:** ^1^ Department of Interventional Neuroradiology Alfred Hospital Melbourne Victoria Australia; ^2^ Faculty of Medicine, Nursing and Health Sciences, Central Clinical School Monash University Melbourne Victoria Australia; ^3^ Department of Radiology Alfred Hospital Melbourne Victoria Australia; ^4^ Department of Neurosurgery Alfred Hospital Melbourne Victoria Australia

**Keywords:** endovascular, flow diverting stent, FRED‐X, intracranial aneurysm, neurointervention

## Abstract

**Background:**

The FRED X flow diverter features antithrombotic surface treatment to reduce thrombogenicity. This study evaluated the safety and efficacy of FRED X in treating intracranial aneurysms in the Australian setting.

**Methods:**

Clinical, procedural and imaging data were retrospectively reviewed for a consecutive series of patients at a single Australian neurovascular centre. Follow‐up imaging was performed with a combination of MRA and IV‐DSA imaging.

**Results:**

A total of 39 consecutive patients (21% presenting with acute rupture) with 52 aneurysms treated with 40 FRED X devices between June 2021 and September 2024 were included in this study. Aneurysms were predominantly saccular (88%) and located in the ICA (82%), with a median size of 5.1 mm (IQR 2.4–8). Satisfactory wall apposition was achieved in 98% of cases. Additional coiling was performed in 20%, and balloon angioplasty in 5%. At a median follow‐up of 28 weeks, complete aneurysm occlusion was achieved in 74% of cases, with adequate occlusion (> 90%) in 86%. Minor adverse events occurred in 10% and major adverse events in 5%. Overall mortality was 5%, exclusively in acute subarachnoid haemorrhage cases.

**Conclusion:**

The FRED X demonstrates favourable safety and efficacy profiles, with high technical success rates and satisfactory occlusion outcomes in an Australian setting. Thrombotic complications were rare, possibly reflecting the benefits of the antithrombotic coating, though larger studies with longer‐term follow‐up are needed for confirmation.

## 1 Introduction

Flow diverters have emerged as a safe and effective endovascular treatment option for wide‐necked intracranial aneurysms designed to occlude aneurysms by utilising dense wire mesh to alter blood flow dynamics, thus promoting intra‐aneurysmal thrombosis and artery remodelling through endothelialisation [1]. However, the high metal content of flow diverters can potentially induce thrombosis, a serious complication that may result in distal emboli, branch vessel occlusion, or even occlusion of the stent itself, causing subsequent ischaemic stroke [2].

The latest generation of flow diverters aims to address this issue by introducing antithrombotic coatings. The previous generation Flow‐Redirection Endoluminal Device (FRED) by Terumo Neuro was a dual‐layer device combining a high‐porosity outer stent for structural support with a low‐porosity inner mesh for flow redirection and has shown promising safety and efficacy in global studies [3]. The newest version, FRED‐X, features the proprietary antithrombotic “X technology” surface treatment designed to reduce thrombogenicity [4].

Early experiences from European studies are emerging with promising results; however, there is a paucity of data on the performance of FRED‐X in the Australian setting [5]. Our single‐centre study aims to provide an Australian perspective on the performance of FRED‐X by reporting on our initial experiences, including its peri‐ and post‐procedural safety profile and efficacy in treating both ruptured and unruptured intracranial aneurysms.

## 2 Methods

A retrospective, observational study was conducted at a specialist neurovascular centre in Australia to evaluate the efficacy and safety of the FRED‐X flow diverter in treating intracranial aneurysms. The study included consecutive patients with both ruptured and unruptured aneurysms who underwent treatment with the FRED‐X device between June 2021 and September 2024.

The study analysed clinical, radiological, and procedural data. Patient demographics, aneurysm characteristics, treatment parameters, and follow‐up imaging were recorded. Standardised scales, such as the O'Kelly‐Marotta (OKM) and Raymond‐Roy occlusion classification (RROC) were used for assessing aneurysm occlusion [6, 7]. The primary outcome of adequate occlusion was defined as complete occlusion or residual neck (OKM C and D and RROC I and II).

Periprocedural technical difficulties, adverse events and mortality were assessed. The severity of adverse events was categorised based on criteria from previous larger European studies: minor adverse events were defined as those resolving within 7 days without clinical sequelae, while major adverse events were characterised by ongoing clinical deficits at 7 days [5].

Data collection adhered to the Strengthening the Reporting of Observational Studies in Epidemiology (STROBE) guidelines and was approved by local ethics committees for retrospective data retrieval.

## 3 Results

All instances of FRED‐X used at our institution during the study period were reviewed. Out of a total of 44 FRED‐X cases, a total of four use cases were excluded. Three of these cases were in the setting of ischaemic stroke mechanical thrombectomy tandem lesion dissection repair. A retreatment case of an inflammatory/malignant giant internal carotid artery (ICA) fusiform aneurysm recurrence in which three overlapping FRED‐X stents were telescoped was also excluded given the very atypical rescue scenario and thus not a fair reflection of typical device performance.

A total of 39 patients, of which 31 were female (79%) with a total of 52 aneurysms, treated with 40 FRED‐X devices, were included in this study. The median age was 57 years (IQR 50–65). Eight patients (21%) presented with subarachnoid haemorrhage due to acute aneurysmal rupture. Another 31 patients (79%) were treated electively for incidental, co‐incidental, or aneurysm recurrence. See Table 1.

**TABLE 1:** Patient characteristics.

Cases	Patients = 39 Cases (FRED‐X deployed) = 40 Aneurysms = 52			
Age (median)	57 (Range 33–80) (IQR 50–65)			
Sex	Female = 31 (79%)			
Clinical presentation	SAH = 8 (21%)	Regrowth/persistent aneurysm = 11 (28%)	Incidental/coincidental = 20 (51%)	
Aneurysm types	Saccular = 46 (88%)	Fusiform = 1 (2%)	Dissecting = 2 (4%)	Blister = 3 (6%)
Aneurysm location	ICA = 41 (79%)	VA = 3 (6%)	BA = 6 (12%)	ACA = 2 (4%)
Median aneurysm size (mm)	5.1 (Range 1–20) (IQR 2.4, 8)			
Median neck diameter (mm)	3.1 (IQR 2.2, 4.6)			
Dome‐to‐neck ratio	1.5 (IQR 1.2, 2)			
Median diameter of parent vessel at aneurysm (mm)	3.6 (IQR 3, 4.3)			

The majority of aneurysms were morphologically saccular (88%) with a median size of 5.1 mm (IQR 2.4–8) and a median dome‐neck ratio of 1.5. Aneurysms were located predominantly at the internal carotid artery (82%), followed by the basilar artery (10%), vertebral artery (6%) and anterior cerebral artery (2%).

Additional coiling was performed in 8 (20%) cases. Balloon angioplasty was performed in 2 (5%) cases to improve wall apposition. Wall apposition was deemed satisfactory in 39/40 (98%) of cases. See Table 2. In one paraophthalmic aneurysm case with tortuous carotid siphon anatomy, the FRED‐X was deployed successfully in a single pass following unsuccessful attempts at effectively deploying a Pipeline Vantage (Medtronic) flow diverter of similar size.

**TABLE 2:** Treatment characteristics.

Antiplatelet regimen	Aspirin + Clopidogrel = 9 (23%)	Aspirin + Prasugrel = 27 (68%)	Aspirin + Ticagrelor = 4 (10%)
Aneurysms treated in respective session	1 = 32 cases	2 = 3 cases	3 = 5 cases
Vessel wall apposition	Satisfactory = 39 (98%)	Incomplete = 1 (2%)	
Additional coiling during case	Yes = 8 (20%)	No = 32 (80%)	
Stent deployment passes	1 = 35 (88%)	2 = 2 (5%)	3 = 3 (7%)
In‐stent balloon angioplasty	Yes = 3 (7%)	No = 37 (93%)	

All elective patients received dual antiplatelet therapy preloading with aspirin in addition to a P2Y12 agent (Prasugrel 68%, Clopidogrel 23%, Ticagrelor 10%). Using VerifyNow assay, documentation of platelet reactivity values to confirm P2Y12 agent efficacy (PRU < 180) was available for all elective cases [8]. Post‐procedural dual antiplatelet therapy practice was consistent across operators. Typically, dual antiplatelet therapy was continued for 6 months, followed by aspirin monotherapy lifelong thereafter.

The FRED‐X stent was used in the setting of acute subarachnoid haemorrhage in eight cases. In five of these cases, the aneurysms treated were dissecting or blister type. Two cases were for saccular type aneurysms where multiple supraclinoid ICA aneurysms were treated with a single stent. One case was an early staged definitive treatment of a paraophthalmic aneurysm following partial coiling.

**FIGURE 1:**
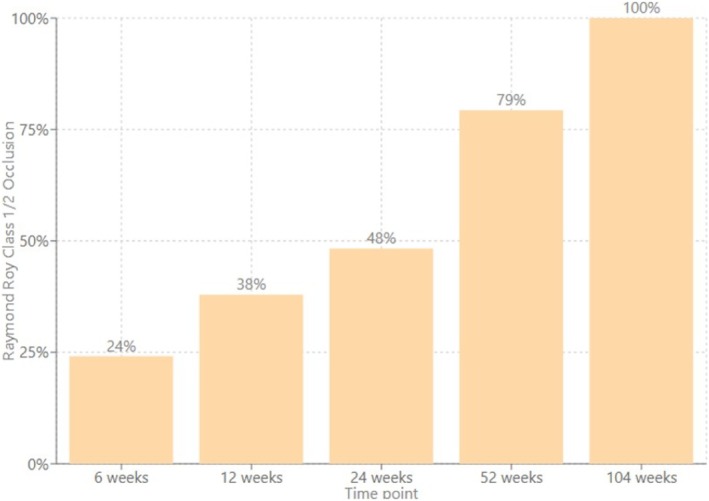
Cumulative percentage distribution of cases that achieved Raymond Roy class 1 or 2 occlusion.

**FIGURE 2:**
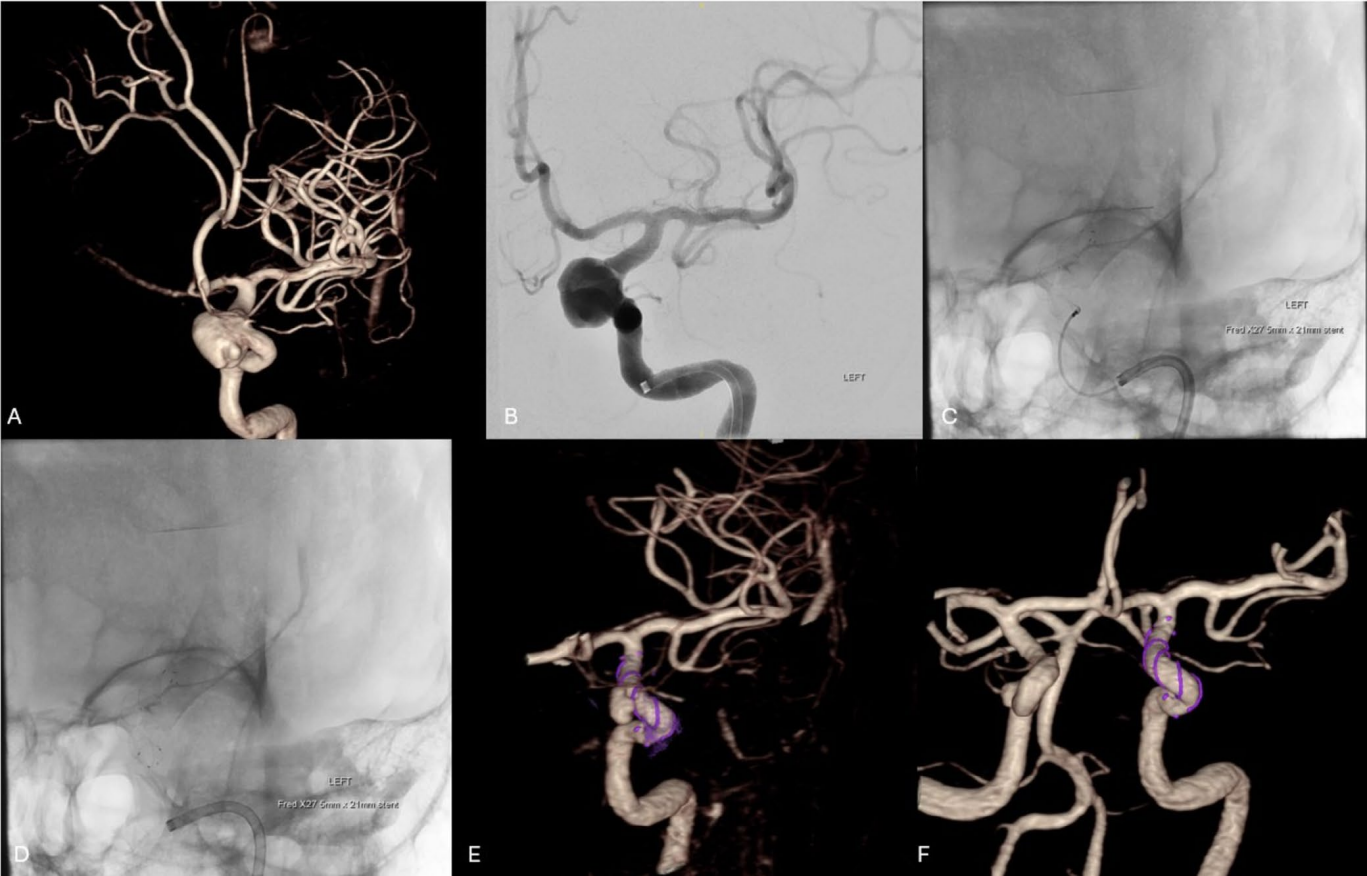
Elective treatment of a 63‐year‐old female with the FRED‐X. 3D reconstruction and working projection angiography revealed an incidental 14 mm left paraophthalmic aneurysm (A, B). A FRED‐X flow diverter was deployed in the parent artery across the aneurysm neck (C, D). IV‐DSA 3D reconstruction 5 and 11 months after treatment shows reduction then occlusion of the aneurysm (E, F).

**FIGURE 3:**
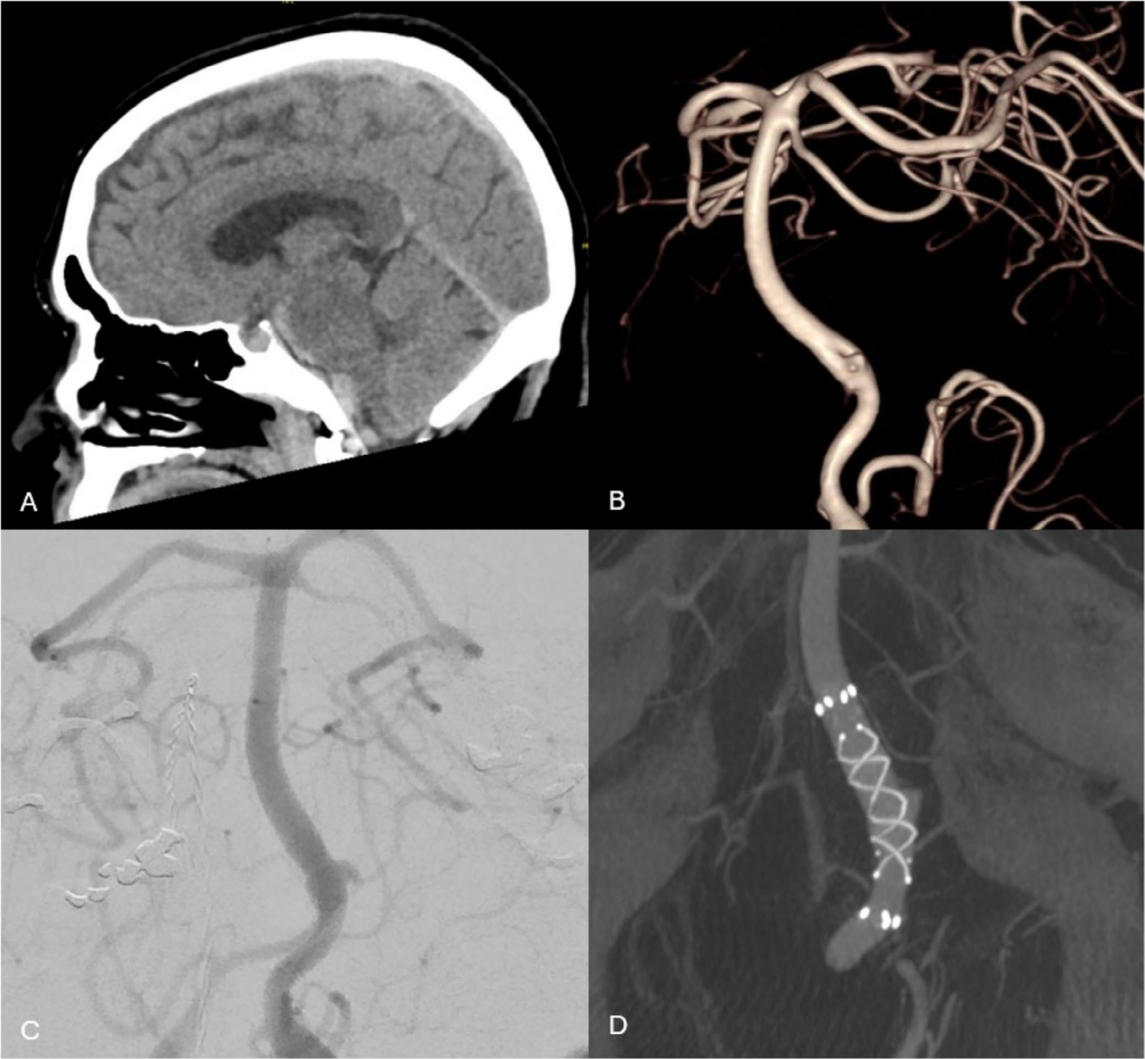
Acute treatment of a 65‐year‐old female with the FRED‐X. The patient presents with WFNS grade 1 subarachnoid haemorrhage with blood in the basal/pontine cisterns and subdural spaces confirmed on CT brain imaging (A). Initial cerebral angiogram was unrevealing. However, a repeat study on day 5 revealed a blister aneurysm at the fenestrated basilar junction (B). AP cerebral angiogram view prior to stenting (C). A FRED‐X flow diverter was deployed across the aneurysm from the basilar artery to the left V4 segment, across the anterior limb of the fenestration. DynaCT shows good wall apposition (D). The patient went on to make an excellent recovery.

Of the 33 cases with available post‐treatment follow‐up imaging, the median follow‐up time frame of this cohort was 28 weeks (IQR 7–78). Seven cases did not have routine follow‐up imaging due to the recency of the procedure at the time of data collection or death during the acute admission. At the latest neuroimaging follow‐up using DSA, IV‐DSA, or MRA modality, complete aneurysm occlusion was found in 31 (74%) aneurysms, residual neck in 5 (12%) aneurysms, and 6 (14%) aneurysms persisted. Table 3. Therefore, taking a threshold of > 90% aneurysm occlusion, the aneurysm was deemed secured in 86% of cases. Of the aneurysms deemed secured, 48% and 79% of these were confirmed to be secured at the 6‐month and 1‐year time points, respectively. See Figure 1. 90% of the followed‐up cases have had stent status assessment, predominantly via IV‐DSA imaging modality. There were no cases of significant in‐stent stenosis, neointimal hyperplasia, stent fish‐mouthing, or braid collapse noted. See Figures 2 and 3 for illustrative case examples.

**TABLE 3:** Occlusion rates and stent status.

Occlusion at latest follow up (*N* = 42 aneurysms)	I: Complete occlusion 31 (74%)	II: Residual neck 5 (12%)	III: Residual aneurysm 6 (14%)
Significant In‐stent stenosis	Yes = 0 (0%)	No = 33 (100%)	
Fish‐mouthing or braid Failure	Yes = 0 (0%)	No = 33 (100%)	
Follow Up imaging modality	90% of follow up cases received IV‐DSA or Catheter DSA follow up imaging. 88% of these were IV‐DSA. 70% of cases have had MRA imaging follow up.		

During follow up, five patients were recorded to have minor adverse events. Table 4. These were as follows: transient dysphasia due to a small infarct within the stent territory, transient homonymous hemianopia due to contrast encephalopathy, and transient contrast induced sialadenitis affecting the parotid glands bilaterally. An instance of acute in‐stent thrombosis was identified intraoperatively for an acute subarachnoid haemorrhage ICA blister aneurysm case for which preprocedural antiplatelet loading was not possible. The FRED‐X stent was deployed following IV aspirin 500 mg loading, along with standard IV heparinisation. IV abciximab was given to manage the thrombus, and ticagrelor was then loaded via a nasogastric tube prior to conclusion of the procedure. Follow up angiograms for vasospasm treatment revealed a widely patent stent construct. Another instance of stent related thrombus occurred during an elective right paraclinoid aneurysm case. There was progressive slowing then occlusion of the jailed right ophthalmic artery due to suboptimal wall apposition. Sceptre XC 4.0 × 11mm balloon angioplasty of the stent at the paraophthalmic level, along with a targeted 1 mg intraarterial tirofiban bolus, ameliorated the issue with successful recanalisation of the ophthalmic artery.

**TABLE 4:** Adverse events.

Adverse event	Minor adverse event (Resolved ≤ 7 days) 5 (12%)	Major adverse event (Clinical deficit > 7 days) 2 (5%)	Total mortality 2 (5%)	Mortality excluding subarachnoid haemorrhage cases 0 (0%)

Two patients were recorded to have major adverse events. One patient suffered an infarct within the stented vascular territory, causing mild contralateral hemiparesis. Another patient had a remote intraparenchymal haemorrhage downstream to the stent within 12 h of stenting. On retrospective review of this specific case, angiographic imaging demonstrated a tiny distal ACA shunting lesion prior to FRED‐X deployment, in the region of a previous ischaemic stroke.

Mortality was confirmed in two patients, both of whom presented with high grade (modified Fisher grade 4, World Federation of Neurosurgical Societies grade 2 and 4) acute subarachnoid haemorrhage with blister aneurysms and subsequently succumbed to severe vasospasm‐related delayed cerebral ischaemia despite securing of the aneurysms with FRED‐X stenting.

## 4 Discussion

FRED‐X, with its novel antithrombotic surface treatment, represents a promising advancement in flow diverter technology for intracranial aneurysm treatment.

Our experience, which is in predominantly smaller saccular aneurysms, demonstrated a complete occlusion rate of 74% at a median 28‐week (IQR 25–80) follow‐up, comparable with previous FRED‐X studies [5, 9]. The progressive occlusion rate over time aligns with the expected intimal remodelling process following flow diversion and underscores the importance of longer‐term follow up.

The safety profile appears favourable, with rates of minor (12%) and major (5%) adverse/complication events comparable to or lower than those reported in larger studies [5, 9, 10]. The 5% total mortality rate reflects deaths in high‐risk cases (acute subarachnoid haemorrhage) rather than device‐related complications. Excluding the use of FRED‐X in acute subarachnoid haemorrhage cases, there were no cases of unexpected mortality.

There is a role for flow diverter stent use in select acute subarachnoid haemorrhage cases, particularly for dissecting or blister type aneurysms for which coiling alone or an intrasaccular device is not an appropriate treatment option. However, balancing stent‐thrombosis risk with concomitant bleeding issues remains a challenge [11, 12]. It is known that the setting of an acute aneurysmal rupture is a particularly inflammatory and prothrombogenic state [13]. At our institution, where possible, preprocedural enteral antiplatelet loading was instituted following placement of external ventricular drains and exclusion of tract haematomas. Platelet reactivity status was assessed using VerifyNow. In cases where preprocedural loading was not possible, typical practice involved IV aspirin 500 mg loading and IV tirofiban bolus then infusion prior to stent deployment. The timing of transition of the IV tirofiban infusion to a P2Y12 agent was guided by the necessity for further open procedures.

An instance of acute stent thrombosis was seen in the setting of an acute subarachnoid haemorrhage case during which the FRED‐X stent was deployed following IV aspirin 500 mg loading and standard IV heparinisation only without a second antiplatelet agent on board. Another instance of stent‐related thrombus occurred during an elective procedure in the setting of suboptimal wall apposition despite appropriate dual antiplatelet therapy loading. At this stage, we lack confidence in regard to the antithrombotic safety of deploying the FRED‐X flow diverter within the intracranial circulation on a single antiplatelet agent. Notably, the absence of significant in‐stent stenosis or neointimal hyperplasia for any case during our follow‐up may reflect the antithrombotic coating technology, though longer‐term studies are needed to confirm this.

FRED‐X appears reliable in deployment, with good rates of satisfactory wall apposition and seldom requires balloon angioplasty. In one case with initial suboptimal wall apposition at the paraophthalmic level, progressive slowing then occlusion of the jailed ophthalmic artery occurred. Although this was rectified with balloon angioplasty and directed intraarterial tirofiban bolus, the incidence underscores the importance of good wall apposition. Wall apposition can be assessed with on‐table diluted contrast Dyna‐CT angiography in cases with uncertainty [14]. The flared end design of the FRED‐X flow diverter appears to encourage good wall apposition with minimal risk of proximal fish mouthing [4]. No instances of stent fish‐mouthing or braid collapse were noted on follow‐up, perhaps related to its unique scaffolded dual layer, flared end design. However, the end tynes offset the working flow diverting length of the stent by 2–3 mm on each end, which can complicate deployment in confined spaces.

Concomitant coiling via a jailed microcatheter technique was performed in 20% of cases in aneurysms that tended to be larger, with a median size of 9 mm (range 7–17 mm) and morphologically irregular. The rationale was to reduce the potential risk of delayed aneurysm rupture.

Our centre incorporated intravenous DSA follow up in addition to traditional MR angiography, combining the advantages of each modality to non‐invasively assess aneurysm and stent status at interval timepoints as guided by the treating neurointerventionalist [14]. Routine follow up catheter DSA review of non‐occluded flow diverted aneurysms was not considered except in very select cases where the aneurysm was deemed to be still at significant risk of rupture and further intervention was feasible.

Study limitations which limit the generalisability of our results include the small sample size, single‐centre design, and retrospective nature of the data.

In conclusion, this study provides real‐world insight on the performance of FRED‐X in the Australian clinical setting. Results are comparable with previous studies and suggest the device maintains the efficacy of its predecessors while potentially offering improved safety. As with any new medical device, ongoing research and vigilance are crucial to fully understand its long‐term performance.

## Ethics Statement

Local low risk ethics approval granted by the Alfred Health Ethics Committee.

## Conflicts of Interest

The authors declare no conflicts of interest.

## Data Availability

The data that support the findings of this study are available from the corresponding author upon reasonable request.
